# Hip fracture care in Zimbabwe: a cohort-based health economic analysis

**DOI:** 10.1302/2633-1462.610.BJO-2025-0107.R1

**Published:** 2025-10-03

**Authors:** Nyashadzaishe Mafirakureva, Pauline Chido Ishumael, Tadios Manyanga, Prudance Mushayavanhu, Munyaradzi Ndekwere, Hannah Wilson, Anya Burton, Simon Graham, James Masters, Matthew L. Costa, Rashida A. Ferrand, Celia L. Gregson, Sian M. Noble

**Affiliations:** 1 Sheffield Centre for Health and Related Research, School of Medicine and Population Health, University of Sheffield, Sheffield, UK; 2 Musculoskeletal Research Unit, Translational Health Sciences, Bristol Medical School, University of Bristol, Bristol, UK; 3 The Health Research Unit Zimbabwe, The Biomedical Research and Training Institute, Harare, Zimbabwe; 4 Department of Surgery, Sally Mugabe Central Hospital, Harare, Zimbabwe; 5 Department of Surgery, Midlands State University, Gweru, Zimbabwe; 6 Oxford Trauma and Emergency Care, Nuffield Department of Orthopaedics, Rheumatology and Musculoskeletal Science, University of Oxford, Oxford, UK; 7 Clinical Research Department, London School of Hygiene and Tropical Medicine, London, UK; 8 Royal United Hospital Bath NHS Trust, Bath, UK; 9 Population Health Sciences, Bristol Medical School, University of Bristol, Bristol, UK

**Keywords:** Hip fractures, Economic evaluation, Cost-effectiveness analysis, Low income setting, surgical approaches, acute hip fractures, morbidity, orthopaedic surgeons, Visual Analogue Scale, radiographs, EQ-5D-5L, healthcare professionals, fragility fractures

## Abstract

**Aims:**

Hip fractures are a leading cause of morbidity and mortality worldwide, particularly among older people. While early surgical management improves outcomes compared to non-surgical approaches, high costs of surgery pose significant barriers in low- and middle-income countries. A cost-utility analysis of hip fracture management was undertaken in Zimbabwe, to guide resource allocation and policy.

**Methods:**

Patient-level data were obtained from a prospective cohort of adults aged 40 years and above with acute hip fractures presenting to hospital in Harare (two public; five private) between October 2021 and October 2022. Healthcare resource use and costs in 2023 USD$ were assessed from individual billing data, with imputed values used for missing resources. Health outcomes were measured in quality-adjusted life-years (QALYs). Incremental cost-effectiveness ratios (ICERs), defined as the ratio of incremental costs to incremental QALYs, were estimated using a regression approach. Sensitivity analyses assessed the impact of different assumptions on cost-effectiveness.

**Results:**

The cohort had 190 patients with an average age of 72 years (SD 14.3), and 51% (n = 97) were male; 61% (n = 116) had surgery for their hip fracture. Patients who underwent surgery had 0.17 (95% CI 0.10 to 0.25) additional QALYs and incurred substantially higher healthcare costs: $1,676 (95% CI 730 to 2,621) higher per patient. The ICER for the primary analysis was $9,647/QALY gained. Restricting the analysis to patients who did not experience extensive surgical delays resulted in smaller difference in costs and an ICER of $4,126/QALY gained. The results were sensitive to the exchange rate used to estimate costs.

**Conclusion:**

Although patients who underwent surgery for hip fractures had higher costs, they had better health outcomes in terms of QALYs. Targeted improvements in provision of surgical care, particularly in minimizing surgical delays, could improve both patient outcomes and lower healthcare costs.

Cite this article: *Bone Jt Open* 2025;6(10):1179–1189.

## Introduction

The costs associated with management of hip fractures are substantial across all settings worldwide.^1^ In high-income countries such as the United Kingdom, healthcare costs have been estimated at approximately £2 billion per year (1.5% of total healthcare spending). Including formal and informal care costs increases total expenditure to approximately £4.8 billion.^2^

The population of older adults in Africa is steadily increasing.^3,4^ As people age, the risk of fragility fractures such as hip fractures increases exponentially. Hence, Africa is expected to see the largest proportional rise in fragility fractures globally.^5,6^ Despite this rapid change, very little is known about the costs of hip fracture care in Africa. The only study we identified from an African context (conducted by our group) reported high healthcare costs for managing hip fractures within South Africa’s public healthcare system.^7^

Clinical pathways for treating fractures are complex and vary across countries, depending on healthcare infrastructure and social conditions.^8,9^ In many high-income countries, early surgical intervention and multidisciplinary care are standard due to their proven effectiveness in improving patient outcomes.^10^ In Africa, around half of hip fracture patients do not receive surgery due to cost-related barriers and a lack of orthopaedic resources.^11^

We conducted an economic evaluation using data from a cohort of patients in Zimbabwe.^12^ We recorded healthcare resource use and patient-reported, health-related quality of life associated with hip fracture care, and calculated costs and quality-adjusted life-years (QALYs). We then compared resource use, costs, and QALYs in patients treated with surgery compared with non-surgical management (based on the treatment patients received).

## Methods

The analysis used prospective ‘real-world’ patient-level data from a cohort of patients with acute hip fractures over a 12-month post-fracture follow-up period.^12^ Analyses were conducted from a healthcare sector perspective which considers and counts all ‘formal healthcare sector (medical) costs borne by third-party payers or out-of-pocket costs paid by patients’.^13^ Costs and health outcomes were not assessed beyond a 12-month post-fracture time horizon hence no discounting was applied.

### Population and setting

This prospective cohort study formed part of the Fractures-E3 (Fractures in sub-Saharan Africa: epidemiology, economic impact, and ethnography) research programme, with the protocol previously reported.^12^ Briefly, the study population included males and females aged ≥ 40 years, residing in Harare Province (urban/peri-urban), who presented with a new hip fracture. Zimbabwe, a lower-middle-income country, had a gross domestic product (GDP) per capita of $1,592 in 2023 and healthcare spending of $63 per capita in 2021. Decades of macroeconomic instability, including hyperinflation, resulted in a severely underfunded healthcare system, compounded by a high burden of communicable diseases, particularly HIV and AIDS. Public healthcare infrastructure is strained by shortages of resources and healthcare workers, with low health insurance coverage leaving much of the population relying on out-of-pocket payments.

Eight hospitals (two public and six private) provide all hip fracture care in Harare Province. Only one small private hospital that rarely saw a hip fracture declined to permit onsite data collection. From October 2021 to October 2022, all patients with an incident hip fracture aged ≥ 40 years were identified. Hip fractures were confirmed by radiographs wherever possible, which were reviewed by two orthopaedic surgeons and classified as intracapsular (International Classification of Diseases of the World Health Organization ((ICD)-10 code S72.0), pertrochanteric (ICD-10 code S72.1), and subtrochanteric (ICD-10 code S72.2). When radiographs were unavailable (arising from intermittent X-ray equipment faults, electricity supply, or unaffordability of radiographs), the mechanism of injury, patient symptoms, and examination findings by an orthopaedic surgeon were used to verify a hip fracture.

Consenting patients were followed up for one year. Data on clinical management, resource use, and health-related quality of life were collected at initial admission and at up to four timepoints during follow-up (one, four, six to eight, and 12 months). Following hospital discharge, follow-up visits were conducted in the patient’s home. Telephone follow-up was arranged for patients in distant rural locations.

### Patient characteristics

Of the 190 consenting patients, the mean age was 72 years (SD 14.3), 51% (n = 97) were male, and most (95%, n = 181) identified as Black African, with over half (57%, n = 108) living in urban areas. Only 61% (n = 116) underwent surgery. Most patients (90%, n = 171) were treated at public hospitals. All patients admitted to private hospitals had surgery. Of the 190 patients, 132 (69.5%) completed 12-month follow-up, four (2.1%) withdrew, five (2.6%) were lost to follow-up, and 49 (25.8%) died. A full description of the study population is shown in Table I and in Supplementary Material.

**Table I.** Cohort characteristics by the type of treatment received.

Characteristic	Overall (n = 190)	Operated (n = 116)	Not operated (n = 74)
Mean age, yrs (SD)	71.99 (14.29)	71.30 (14.89)	73.08 (13.31)
Female, n (%)	93 (48.9)	55 (47.4)	38 (51.4)
Black African ethnicity, n (%)	181 (95.3)	107 (92.2)	74 (100.0)
Urban residence, n (%)	108 (56.8)	68 (58.6)	40 (54.1)
Previous fracture, n (%)	23 (12.1)	15 (12.9)	8 (10.8)
Presented to a public hospital, n (%)	171 (90.0)	97 (83.6)	74 (100.0)
Presented to a private hospital, n (%)	19 (10.0)	19 (16.4)	0 (0.0)
**Care pathway, n (%)**			
Direct hospital presentation	120 (63.2)	64 (55.2)	56 (75.7)
Referred from other facilities*	70 (36.8)	52 (44.8)	18 (24.3)
**Type of fracture, n (%)**			
Intertrochanteric	66 (38.6)	35 (34.3)	31 (44.9)
Intracapsular	79 (46.2)	45 (44.1)	34 (49.3)
Subtrochanteric	26 (15.2)	22 (21.6)	4 (5.8)
Had surgery within 15 days of admission	57 (30.3)	57 (50.0)	0 (0.0)
**End of 12-month follow-up status, n (%)**			
Alive	132 (69.5)	90 (77.6)	42 (56.8)
Withdrawn	4 (2.1)	2 (1.7)	2 (2.7)
LTFU	5 (2.6)	3 (2.6)	2 (2.7)
Dead	49 (25.8)	21 (18.1)	28 (37.8)

* Other facilities included general practitioner, healthcare centre, clinic, or hospital.

LTFU, lost to follow-up.

Overall, 72 (37.9%) patients had complete data for both costs (all resource use items for all five assessment timepoints), and QALYs (all five EuroQol five-dimension five-level questionnaire (EQ-5D-5L) dimensions for all five assessment timepoints) over the 12-month follow-up period (Supplementary Material). This included 25/74 (33.8%) patients managed non-surgically and 47/116 (40.5%) who underwent surgery.

### Resource use measurement and valuation

Resource use and costs included care received before initial hospital admission, inpatient care (initial and subsequent admission), outpatient follow-up, and any community-based care. Data collection identified individual patient-specific care pathways for hip fracture management; the data collection tools were informed by reviews of a sample of inpatient forms/hospital bills and validated with orthopaedic specialists.

Initial in-hospital resource use data were primarily abstracted from patients’ hospital bills provided by hospital accountants. Data spanned Emergency Department (ED) presentations, ward stay/bed stay, theatre/operation, blood and blood product use, investigations, consumables, specialist services (e.g. physiotherapy), and medications, including those provided on discharge.

Healthcare resource use for care received before and after initial hospital admission was collected through a bespoke researcher-administered questionnaire, completed with the patient and/or their caregiver. Data covered outpatient healthcare facility visits, hospital readmissions, home visits by healthcare professionals, medications, home adaptations or modifications, specialist equipment costs, and assistive devices (e.g. walking aids). Whenever readmission was reported, detailed bills outlining the care provided were retrieved from the relevant hospital.

Resources used during initial and subsequent in-hospital care were valued using unit or total costs provided with the medical bills. Resource use for care received before initial presentation and other post-discharge resource use was valued using patient and/or caregiver-reported costs. Resource use for patients who died before a particular follow-up timepoint was considered missing for that timepoint. Resource use for all subsequent timepoints was assigned a zero value.

Although the Zimbabwean dollar (ZWL$) was the official currency from 2019,^14^ a multicurrency economy has persisted for more than a decade, so costs collected could be in ZWL$ or USD$. Several foreign currency exchange rates existed, including: 1) the Official/Interbank Rate set by the Reserve Bank of Zimbabwe (RBZ); 2) the Auction Rate determined by RBZ auctions for companies needing US$ for imports; and 3) the Parallel/Informal Market Rate, set by unregulated traders, often higher than the official rates due to market forces. Costs recorded in ZWL$ were converted to USD$ using the three prevailing exchange rates (tracked daily for the duration of data collection).^15^ The primary analysis used the Official/Interbank Exchange Rate, as advised by local experts to align with rates used by study hospitals, with Auction and Informal Market exchange rate results provided in the Supplementary Material. Exchange rates were matched to admission or data collection dates to convert all recorded ZWL$ costs to USD$. Costs were adjusted for inflation to 2023 prices using USA inflation rates obtained from the World Bank,^16^ to account for hyperinflation.

### Health outcome measurement and valuation

The primary economic outcome measure was QALYs derived from responses obtained using the EQ-5D-5L and EuroQol Visual Analogue Scale (EQ-VAS) instruments.^17^ At baseline, patients were asked to recall their health status immediately prior to their hip fracture. During follow-up, patients reported their health status for that day.

Patients’ EQ-5D-5L responses were mapped to the Zimbabwean crosswalk value set^18^ to derive corresponding utility scores. Patients who died before reaching a specific follow-up point were assigned a utility value of 0 for that and all subsequent follow-up points. QALYs were calculated for each patient using the area-under-the-curve approach.^19^ In this approach, the mean utility value for two consecutive timepoints is multiplied by the time between the two timepoints expressed in years, after which the results of all periods are summed to calculate total QALYs.

### Statistical analysis

Plans for this analysis were previously described,^12^ and prespecified in a health economic analysis plan (unpublished). Mean resource use, SD, and the number of patients providing data for each resource category were calculated for patients who received surgery and those who did not. Patient-level costs for individual healthcare resource use items were summed to compute total costs per patient at each timepoint. Unadjusted mean total costs and associated bias-corrected and accelerated bootstrapped 95% CIs were calculated. Mean utility per patient was estimated for surgical and non-surgical groups at baseline and all follow-up timepoints. Total unadjusted mean (95% CI) QALYs per patient were estimated. Resource use for ED visits, ward/bed stays, and operations was drawn from medical records when not detailed on hospital bills. Missing data on implant use and staff surgery time (for surgeons, nurses, and anaesthetists) were estimated in consultation with orthopaedic specialists. Unit costs for emergency presentations, bed stays, and operations were estimated using average costs from patients with complete records, with adjustments for hospital type (private or public). Patient and/or caregiver-reported costs were used to value readmission costs if the hospital bill could not be retrieved (12/26 readmissions). Market-based unit costs for missing data on implants, staff time, radiological examinations, and mobility aids (e.g. sticks, frames, or crutches) were gathered from local private providers.

A descriptive analysis of missing cost and utility data was performed to establish the patterns and type of missingness, and missing data were assumed to be missing at random (MAR) and imputed based on observed data using multivariate imputation by chained equations (MICE) with predictive mean matching (PMM),^20^ stratified by surgical and non-surgical treatment. To maximize the amount of available data at each timepoint costs were imputed separately for individual cost categories, while utility data were imputed as a single aggregate value for health utilities. The imputation models included age at admission, sex, ethnicity, hospital of admission, fracture type and observed outcomes (utility and costs) as predictors. Complete follow-up data were available for 34% of patients; thus, 70 complete datasets were generated as recommended.^21^ The imputation procedure was implemented using the *mice* R package.^20^ The imputed datasets were analyzed using seemingly unrelated regressions (SUR), using the R package *systemfit*,^22^ to calculate the adjusted mean differences in total costs and QALYs between the two treatment groups. SUR allows for the correlation between random errors associated between the individual-level costs and QALYs by simultaneously modelling two separate standard linear regression equations (i.e. one for total costs and one for QALYs). The QALY regression model was adjusted for treatment arm, age, sex, ethnicity, residence (urban/rural), type of hospital (public/private), previous fracture, fracture type, presentation pathway (direct/referred), and baseline utility. The cost regression model included the same variables except baseline utility. Estimates obtained for each imputed dataset (mean and 95% CI for costs and QALYs) were combined using Rubin’s Rule,^21^ and used to calculate the incremental cost-effectiveness ratios (ICERs) by dividing the difference in adjusted costs by the difference in adjusted QALYs. Statistical uncertainty surrounding the estimated incremental cost and QALYs was estimated using non-parametric bootstrapping (1,000 replications). The impact of assumptions was evaluated through sensitivity analyses: 1) using different currency exchange rates to estimate costs; 2) restricting to patients treated in public hospitals, as all patients in private hospitals had surgery and incurred substantially higher costs; 3) excluding imputed implant and surgical staff costs, which were based on assumptions and potentially overestimated costs; 4) restricting the analysis to patients who had surgery within 15 days of admission; and 5) using simple mean imputation to utility values, where missing data points were replaced with the mean of observed values. To account for potential underestimation, the imputed values were inflated by 10%.

## Results

### Resource use

Overall, resource use was similar for the two treatment pathways across most categories (Table II, Supplementary Material). However, patients who received surgery had a higher mean number (5.1 vs 4.1) of investigations initiated in the ED. The mean length of hospital stay was 24.6 days (SD 18.9) in those managed non-surgically, slightly longer than the 23.1 days (SD 19.3) in those who received surgery. Although overall numbers were low, those having surgery had greater use of inpatient investigations (6.7 vs 5 investigations), and non-theatre consumables (4.2 vs 1.2 consumables) per patient.

**Table II.** Mean resource use at baseline by type of treatment received.

Resource	Operated (n = 116)		Not operated (n = 74)*	
	Number of patients, n (%)†	Resource use, mean (SD)	Number of patients, n (%)†	Resource use, mean (SD)
**Care before coming to the hospital**				
Healthcare facility visit	116 (100)	0.4 (0.5)	74 (100)	0.4 (0.5)
**In-hospital patient stay (initial admission)**				
ED consultation	116 (100)	1.1 (0.2)	74 (100)	1 (0.2)
ED consumables	116 (100)	1.2 (2.9)	74 (100)	1 (2.7)
ED investigations	116 (100)	5.1 (3.6)	73 (98.6)	4.1 (3.6)
ED medicines	116 (100)	0.7 (1.2)	74 (100)	0.8 (1.5)
Ward stay/bed stay, days	116 (100)	23.1 (19.3)	74 (100)	24.6 (18.9)
Initial consultation in ward	116 (100)	0.4 (0.6)	74 (100)	0.5 (0.6)
Subsequent consultation in ward	116 (100)	0.8 (1.7)	74 (100)	1 (2.1)
Theatre/operation fee	112 (96.6)	1.04 (0.2)	74 (100)	0.08 (0.3)
Theatre/operation extra fee	95 (81.9)	0.04 (0.2)	71 (95.9)	0 (0)
Theatre/operation procedure	112 (96.6)	1.02 (1)	74 (100)	0.23 (0.6)
Orthopaedic implant	116 (100)	0.44 (0.8)	74 (100)	0 (0)
Theatre specialized equipment uses	116 (100)	0.03 (0.2)	74 (100)	0 (0)
Anaesthetic gases, mins	84 (72.4)	35.8 (152.6)	71 (95.9)	0 (0)
Recovery room use, days	107 (92.2)	0.46 (0.5)	73 (98.6)	0.04 (0.2)
Orthopaedic surgeon	116 (100)	0.19 (0.4)	74 (100)	0 (0)
Anaesthetist	116 (100)	0.19 (0.4)	74 (100)	0 (0)
Scrub nurse	116 (100)	0.19 (0.4)	74 (100)	0 (0)
Theatre consumables	99 (85.3)	1.6 (3)	74 (100)	0 (0)
Blood & blood products, units	114 (98.3)	0.1 (0.5)	74 (100)	0 (0)
Intensive care unit, days	116 (100)	0.14 (0.9)	74 (100)	0 (0)
High dependency unit, days	115 (99.1)	0.38 (1.5)	73 (98.6)	0 (0)
Inpatient medicines	116 (100)	0.7 (1.7)	74 (100)	0.2 (0.4)
Inpatient investigations	116 (100)	6.7 (7.8)	74 (100)	5 (6.2)
Non-theatre consumables‡	116 (100)	4.2 (4.1)	74 (100)	1.2 (2.3)
Specialist service sessions	116 (100)	0.1 (0.4)	74 (100)	0.1 (0.3)

* Operation status (Operated or Not operated) relates to hip fracture management, but some patients had surgical procedures that were not directly related to hip fracture management (e.g. surgical traction, wound debridement, exploratory laparotomy, patellectomy).

† Some numbers of patients are less than the total because the quantity of those resources for some patients could not be ascertained as they were not disaggregated/itemized, or some data were missing.

‡ Some may include theatre consumables since they were not disaggregated/itemized.

ED, Emergency Department.

### Costs

The mean total unadjusted cost for patients who had surgery was considerably higher at $3,887 (95% CI 2,805 to 5,271) compared with $1,461 (95% CI 1,028 to 1,958) for those who did not (Table III). The index hospital stay accounted for the largest cost difference, with a mean of $3,823 (95% CI 2,843 to 4,826) in surgically managed patients compared with $952 (95% CI 712 to 1,202) in those managed non-surgically. Patients who received surgery had higher follow-up admission costs of $197 (95% CI 24 to 436) compared with $108 (95% CI 1 to 293) in those managed non-surgically. Other follow-up costs, such as facility visits, home visits by healthcare professionals, and equipment, were generally higher in surgically treated patients.

**Table III.** Mean unadjusted total costs aggregated over the main resource categories over the 12-month follow-up period. All costs are presented in 2023 USD$.

Resource	Operated		Not operated	
	N*	Mean cost, USD$ (95% CI)	N*	Mean cost, USD$ (95% CI)
Pre-admission	116	118 (94 to 146)	74	89 (66 to 113)
First admission	116	3,823 (2,843 to 4,826)	74	952 (712 to 1,202)
Follow-up readmission(s)	70	197 (24 to 436)	31	108 (1 to 293)
Facility visit, e.g. outpatient clinic appointment(s)	69	133 (95 to 179)	31	52 (27 to 83)
Home visit(s) by healthcare professionals	69	6 (2 to 10)	31	0 (0 to 0)
Home adaptation(s)	69	21 (2 to 52)	31	3 (0 to 10)
Equipment/walking aids	69	89 (71 to 108)	31	95 (50 to 159)
Medicines	69	46 (29 to 69)	31	36 (24 to 51)
Total cost, USD$	69	3,887 (2,805 to 5,271)	31	1,461 (1,028 to 1,958)

* Shows the number of patients with data available to calculate the mean and 95% CI. The variation in the number of patients reflects missing data for each resource category. Overall, the number of patients with complete data was 100 (53%). Reasons for incomplete data were: patients had died (26%, n = 49), missed appointments (19%, n = 37), and consent withdrawal (2%, n = 4).

### QALYs

**Fig. 1:**
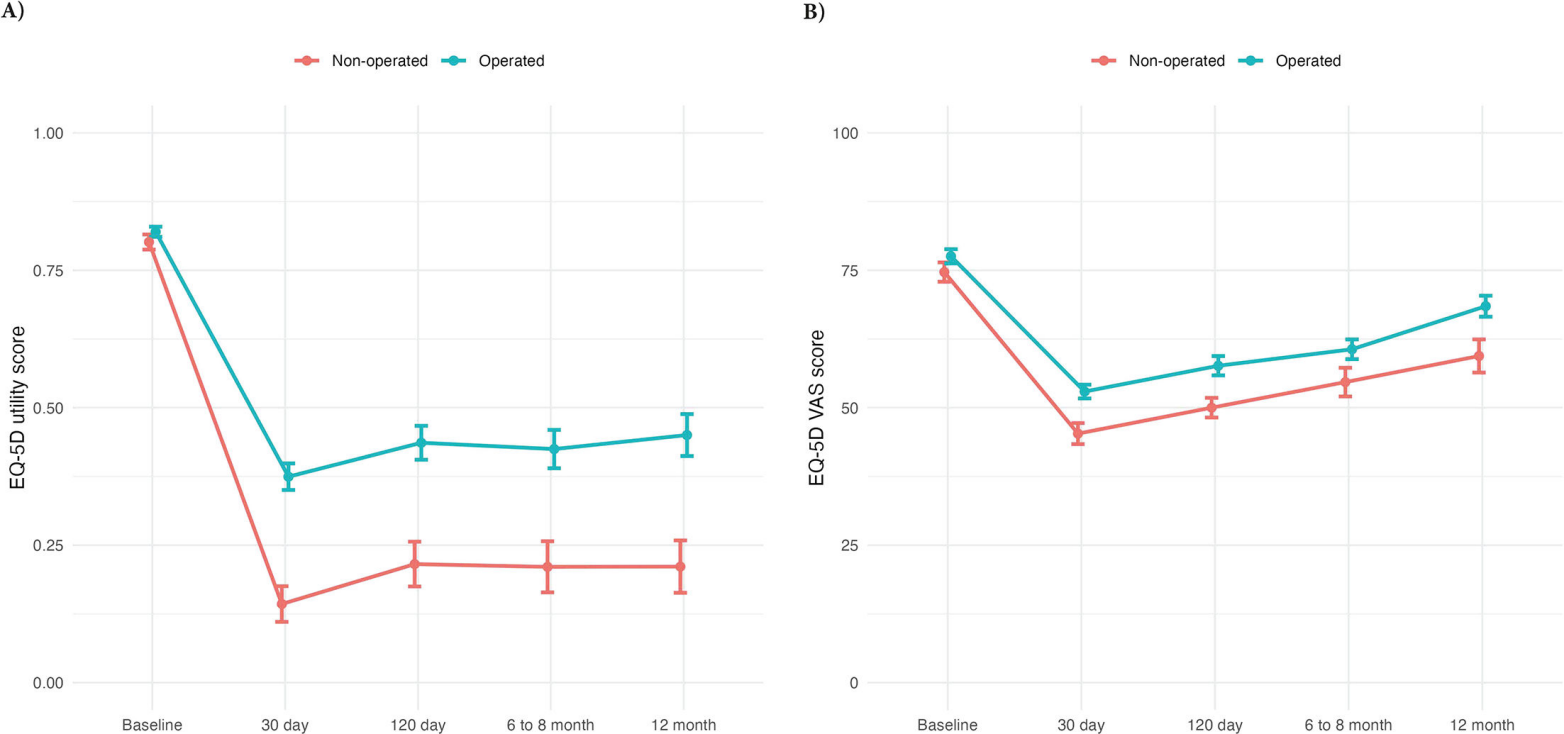
Unadjusted mean a) utilities and b) EuroQol Visual Analogue Scale (EQ-VAS) scores per patient by type of treatment received at baseline and each timepoint over the 12-month follow-up period. EQ-5D, EuroQol five-dimension questionnaire.

The mean EQ-5D utility values and EQ-VAS scores were similar at baseline in patients who did and did not receive surgery (Figure 1). Over 12-month follow-up both measures were significantly lower in patients managed non-surgically, indicating worse quality of life, with more pronounced differences for utility values. Similar one-year patterns showed a sharp decline by 30 days, an improvement at 120 days, and a plateau afterwards. Both measures remained below the baseline levels for the entire follow-up. Non-surgically managed patients had lower unadjusted total mean QALYs (0.24, 95% CI 0.17 to 0.31) compared to those who had surgery (0.44, 95% CI 0.38 to 0.5).

### Economic evaluation

**Fig. 2:**
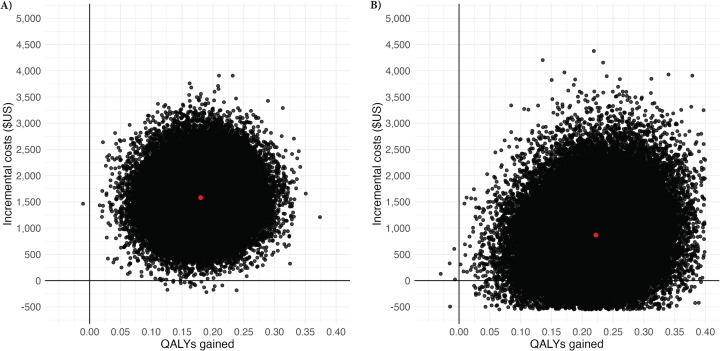
Cost-effectiveness planes showing the differences in costs (y-axis) and quality-adjusted life-years (QALYs) gained (x-axis) of surgical treatment of hip fracture, compared with non-surgical treatment based on 1,000 bootstrap samples. Panel A (left) shows base case results based on covariate adjusted regression using the official/interbank exchange rate, and panel B (right) shows results when the analysis is restricted to patients who had surgery within 15 days of admission. The red dot represents the mean incremental costs and QALYs gained.

After adjusting for baseline covariates, patients who had surgery incurred $1,676 (95% CI 730 to 2,621) higher costs and had 0.17 (95% CI 0.1 to 0.25) more QALYs compared to those managed non-surgically (Table IV). The distribution of differences in costs and QALYs for surgically managed patients relative to those managed non-surgically are shown on Figure 2. The ICER for patients who had surgery in comparison to those who did not was $9,647/QALY gained.

**Table IV.** Total adjusted quality-adjusted life-years (QALYs), costs, and cost-effectiveness analysis. All costs and QALYs were estimated using regression models adjusted for age, sex, ethnicity, residence (urban/rural), type of hospital (public/private), previous fracture, type of fracture, presentation pathway (direct/referred), and baseline utility.

Variable	N	Total costs (95% CI)	QALYs (95% CI)	ICER (USD$ per QALY gained)
**Base case**				
Operated	116	3,862 (3,015 to 4,710)	0.42 (0.37 to 0.48)	
Not operated	74	2,187 (1,441 to 2,932)	0.25 (0.18 to 0.31)	
Difference		1,676 (730 to 2,621)	0.17 (0.09 to 0.25)	9,647
**Patients who had surgery within 14 days of admission only**				
Operated	57	3,634 (2,383 to 4,884)	0.44 (0.36 to 0.53)	
Not operated	74	2,765 (1,793 to 3,737)	0.23 (0.16 to 0.3)	
Difference		869 (-409 to 2,146)	0.21 (0.1 to 0.32)	4,126
**Theatre costs not imputed**				
Operated	116	3,232 (2,510 to 3,954)	0.43 (0.38 to 0.48)	
Not operated	74	1,678 (1,093 to 2,262)	0.25 (0.18 to 0.32)	
Difference		1,554 (643 to 2,465)	0.18 (0.1 to 0.25)	8,864
Simple mean imputation for utilities*				
Operated	116	3,852 (3,006 to 4,699)	0.43 (0.39 to 0.48)	
Not operated	74	2,172 (1,429 to 2,916)	0.25 (0.2 to 0.31)	
Difference		1,680 (733 to 2,627)	0.18 (0.11 to 0.25)	9,393
**Public hospital patients only**				
Operated	97	2,852 (2,145 to 3,559)	0.42 (0.37 to 0.48)	
Not operated	74	1,119 (711 to 1,527)	0.25 (0.18 to 0.31)	
Difference		1,733 (804 to 2,663)	0.17 (0.09 to 0.25)	10,035
**Auction exchange rate**				
Operated	116	2,971 (2,457 to 3,485)	0.43 (0.38 to 0.48)	
Not operated	74	2,000 (1,495 to 2,505)	0.25 (0.18 to 0.32)	
Difference		971 (408 to 1,534)	0.18 (0.1 to 0.25)	5,535
**Parallel market exchange rate**				
Operated	116	2,355 (1,891 to 2,818)	0.42 (0.37 to 0.48)	
Not operated	74	1,705 (1,244 to 2,167)	0.25 (0.18 to 0.31)	
Difference		649 (266 to 1,033)	0.17 (0.09 to 0.26)	3,733

* Missing data points were replaced with the mean of observed values and the imputed values were inflated by 10% to account for potential underestimation. To account for potential underestimation, the imputed values were inflated by 10%. All costs are presented in 2023 USD$.

ICER, incremental cost-effectiveness ratio; QALY, quality-adjusted life-year.

### Sensitivity analyses

Restricting the analysis to patients who had surgery within 15 days of admission (Figure 2, Table IV) reduced the difference in costs for surgical compared with non-surgical management to $869 (95% CI -409 to 2,146) resulting in an ICER of $4,126/QALY gained and indicating that earlier surgery reduced costs. Focusing only on patients treated in public hospitals resulted in much lower costs in the surgical treatment group; however, the differences in costs and QALYs remained similar, resulting in an ICER of $10,035/QALY gained. Excluding imputed theatre costs resulted in a slight decrease in the difference in costs of $1,554 (95% CI 643 to 2,465) and an ICER of $8,864/QALY gained. Applying alternative exchange rates in the calculation of costs resulted in much smaller differences in costs resulting in lower ICERs of $5,535 and $3,733/QALY gained for the Auction and Parallel market exchange rates, respectively.

**Fig. 3:**
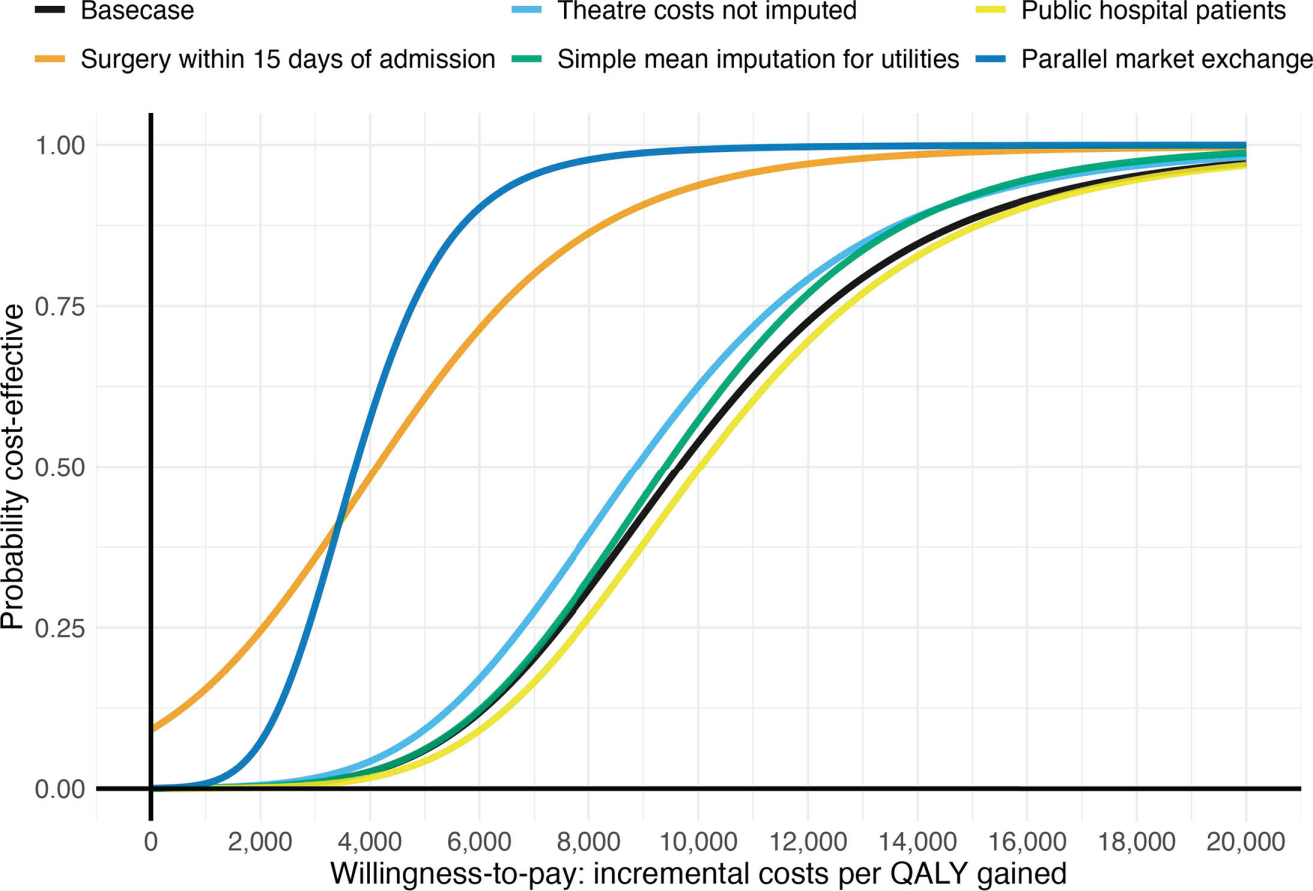
Cost-effectiveness acceptability curves showing the probability of surgical treatment being cost-effective (y-axis) compared to non-surgical treatment over a range of different decision-maker’s cost-effectiveness threshold choices (x-axis). All costs are presented in 2023 USD$.

Surgical treatment for hip fractures demonstrated higher probabilities of being cost-effective at lower willingness-to-pay thresholds in analyses that focused on patients who had surgery within 15 days of admission and when alternative exchange rates were applied (Figure 3).

## Discussion

Our results confirm that surgical treatment of patients with hip fractures substantially improves health-related quality of life compared with non-surgical treatment. These QALY gains equate to an extra 58 days of perfect health within one year. However, these benefits are necessarily accompanied by a higher use of healthcare resources, resulting in significantly higher costs. Non-surgical management was provided exclusively in public hospitals. While non-surgical management appears cheaper, it would be difficult to justify given the adverse impact on QALYs.

Although surgical costs are typically higher initially, in many healthcare systems they are offset by reduced hospital care costs due to shorter hospital stays and lower complication rates.^23^ However, given the substantial delay in patients receiving surgery in Zimbabwe, particularly in the public sector, the overall length of stay for both surgically and non-surgically treated patients was similar, with very long hospital stays in both groups, thus diminishing this expected cost offset. A sensitivity analysis that excluded patients who had surgery after 15 days of admission resulted in a 57% reduction in the ICER, demonstrating the consequences of prolonged surgical delays. Even 15 days would be considered severely delayed in many healthcare economies.^22^ Our recent national service availability and readiness survey in Zimbabwe identified widespread deficits in fracture care provision, including workforce shortages and lack of basic surgical equipment, which is likely to contribute to delays.^24^ Our qualitative research has identified marked challenges experienced by families who can take weeks to mobilize the capital necessary to purchase expensive (out-of-pocket) surgical implants; often this money cannot be found, and therefore surgery cannot be provided.^24^

Our findings also highlighted the sensitivity of estimated costs to fluctuations in, and the choice of, currency exchange rates. Results can vary significantly with the exchange rate used; an intervention may seem cost-effective at the unofficial market rate, but not at the formal exchange rate. This highlights the necessity of carefully considering economic factors when interpreting economic evaluations in resource-limited environments which are marked by economic uncertainty.

Given that Zimbabwe, as in other low- and middle-income countries, lacks an explicitly defined cost-effectiveness threshold, analysts often rely on benchmarks such as the GDP per capita.^25^ The determination of an appropriate threshold ultimately rests with policymakers; GDP per capita-based thresholds have been criticized for their inability to correctly represent the opportunity cost.^26^ Therefore, we only present cost-effectiveness acceptability curves which can assist policymakers in evaluating the probability of cost-effectiveness at their preferred thresholds, with higher thresholds favouring surgical treatment.

Globally, few studies have directly evaluated the economics of surgical compared with non-surgical treatment for hip fractures. This is unsurprising as, in high-income healthcare systems, surgery is widely regarded as the standard of care given its proven effectiveness in improving patient outcomes.^10^ For example, a systematic review found no studies directly comparing healthcare costs and quality of life between surgical and non-surgical treatment groups.^27^ An analysis from the Netherlands comparing operative and nonoperative management in frail institutionalized patients with a hip fracture found an ICER of €76,912/QALY gained.^28^ This high ICER was attributed to the limited quality of life gains in older, frail patients nearing the end of life. In contrast, a USA-based modelling study reported that surgery generated lifetime societal benefits exceeding the direct medical costs by over $60,000 for adults with displaced hip fractures.^23^ While not directly comparable to the present study, these global insights highlight the need for targeted improvements in surgical care for hip fractures, which could ultimately enhance patient outcomes and healthcare system efficiency in Africa.

The primary limitation of our analysis is its reliance on observational data, as opposed to randomized trial data. We adjusted for several potential confounders and baseline utility was similar between the two groups of participants, but residual confounding may remain. However, our data represent actual clinical practices and thus serves as an important natural experiment providing real-life evidence. This provided insights into care pathways, resource use, costs, and health outcomes, enhancing the reliability of the findings in this setting. Our analysis over one year only provides insights into the short- to medium-term costs and outcomes but does not capture potential long-term consequences. The study could not assess delayed complications, long-term functional changes, or other late-emerging effects. Although our results indicate comparable costs at 12 months (Supplementary Material), they show significantly lower QALYs for non-surgically treated patients compared to those who were surgically managed, suggesting that surgical treatment may become more cost-effective over the long term. Consequently, while our findings highlight immediate post-treatment outcomes, extended follow-up studies would provide a more comprehensive evaluation of long-term costs and outcomes.

The cost analysis primarily relied on hospital billing data, which can be incomplete and may omit relevant resources associated with patient care, leading to potential underestimation of true healthcare costs. Efforts were made to minimize this by triangulating different data sources. Missing billing data were imputed using information from patients’ medical records, especially for major resource categories like Emergency Department visits, ward/bed stays, and operations. Additionally, orthopaedic specialists in Zimbabwe were consulted to estimate resource use not captured on bills, such as staff time during surgery and implant costs. However, the assumptions that these costs were not included in patients’ hospital bills may have inadvertently inflated reported costs for surgical patients. Despite these measures, uncertainty and potential bias remain due to reliance on imputation. Furthermore, although hospitals in the study primarily relied on the same tariff schedule for billing, variations in how these tariffs were applied, along with different billing systems and coding practices, may have led to inconsistencies in the recording of resource use and cost data. Billing data often reflect discounted rates, insurance negotiations, or adjustments specific to certain groups, such as those on government aid, which were not accounted for in this analysis. This variability complicates the standardization of unit costs across facilities. Notably, these data were primarily collected for administrative rather than economic evaluation purposes. For example, aggregate resource use was recorded for some patients, such that only total costs were documented, preventing derivation of specific quantities used or their unit costs. Additionally, some resource use and costs unrelated to hip fracture care may have been included, complicating cost comparisons at different levels of resource use.

To the best of our knowledge, this study is the first in Africa to evaluate both the costs and outcomes of hip fracture care. The findings confirm that surgical treatment is associated with substantial costs, but also highlight the potential for surgery to significantly improve patient outcomes. In a region where such economic analyses are scarce, these findings offer valuable insights for shaping healthcare policy and resource allocation strategies.

The high costs associated with treating hip fractures highlight the urgent need for comprehensive interventions focused on both fracture prevention and optimizing patient care when fractures occur. Preventative strategies, such as fracture risk assessment, and antiresorptive treatments are potential approaches to mitigate added economic burdens on already strained healthcare systems.^22^ Additionally, when fractures do happen, improving the efficiency and quality of patient care, such as through timely surgery, enhanced postoperative rehabilitation, and streamlined care pathways, can help optimize outcomes and reduce overall costs.^22^

Ongoing research is crucial to further refine economic evaluations for hip fracture interventions and provide robust evidence for decision-making in resource-limited settings. Further work should focus on improving cost estimation methods, and assessing broader, long-term consequences. Such evidence will better equip healthcare stakeholders to balance the trade-offs between patient outcomes and cost management, ultimately improving the quality of care for hip fracture patients in low- and middle-income countries.

**Take home message**

- Hip fractures impose a growing burden in low- and middle-income countries, where access to surgery is often constrained by high costs.

- Evidence from this study shows that surgical management improves quality-adjusted life years despite higher healthcare costs in Zimbabwe.

- Reducing surgical delays could further improve outcomes and lower costs, supporting investment in surgical capacity in resource-limited settings.

## Data Availability

Data are available upon reasonable request. Researchers can access participant level data via data.bris, provided ethical approvals are in place.
